# The 2D Structure of the* T. brucei* Preedited RPS12 mRNA Is Not Affected by Macromolecular Crowding

**DOI:** 10.1155/2017/6067345

**Published:** 2017-06-18

**Authors:** W.-Matthias Leeder, Stephan Voskuhl, H. Ulrich Göringer

**Affiliations:** Department of Molecular Genetics, Darmstadt University of Technology, Darmstadt, Germany

## Abstract

Mitochondrial transcript maturation in African trypanosomes requires RNA editing to convert sequence-deficient pre-mRNAs into translatable mRNAs. The different pre-mRNAs have been shown to adopt highly stable 2D folds; however, it is not known whether these structures resemble the in vivo folds given the extreme “crowding” conditions within the mitochondrion. Here, we analyze the effects of macromolecular crowding on the structure of the mitochondrial RPS12 pre-mRNA. We use high molecular mass polyethylene glycol as a macromolecular cosolute and monitor the structure of the RNA globally and with nucleotide resolution. We demonstrate that crowding has no impact on the 2D fold and we conclude that the MFE structure in dilute solvent conditions represents a good proxy for the folding of the pre-mRNA in its mitochondrial solvent context.

## 1. Introduction

The folding of RNA molecules into compact, native structures or ensembles of structures is dictated by a set of first-principle physicochemical forces [1]. One of which is charge compensation to overcome the electrostatic repulsion of the negatively charged phosphodiester backbone [2–4]. Mono- and divalent metal ions at low to high millimolar concentrations contribute to this task [5] and the effects of metal ion-radius and charge density have been studied in detail [6–8]. Next to metal ions, metabolites, polyamines, and osmolytes have been shown to modulate RNA structure [9–13] as well as high concentrations of macromolecules, which can occupy up to 30% of the total volume of a cellular compartment. This generates so-called “crowded” solvent conditions [14–16], which in general stabilize RNA 2D and 3D structures through the excluded volume effect and entropy perturbation of the folding landscape [5, 17, 18].

RNA editing describes a posttranscriptional modification reaction of mitochondrial pre-mRNAs that is characterized by the site-specific insertion and deletion of exclusively U-nucleotides (for a review, see [19]). The reaction takes place within the single mitochondrion of trypanosomes, which represents the most crowded intracellular environment of eukaryotic cells. Intramitochondrial macromolecular concentrations reach up to 560 g/L [20, 21]. Editing is catalyzed by a macromolecular protein complex, the 20S editosome [19], which interacts with 18 mitochondrial pre-mRNAs as substrates in the processing reaction. The different transcripts encode subunits of the mitochondrial electron transport and oxidative phosphorylation chains and have been characterized by several unusual features: first, the majority of pre-mRNAs lack substantial sequence information (on average 45%); hence they require RNA editing to be converted into translatable mRNAs. Second, the different pre-mRNAs are typified by an extraordinarily high G-content (34%), which in two-thirds of the cases are clustered in tracts of G-nucleotides (2 ≤ G ≤ 8). Third, in vitro chemical probing studies revealed that the different pre-mRNAs adopt extraordinarily stable 2D- structures approaching the stability of structural RNAs [22, 23]. Next to canonical base-pairing, they contain pseudoknots and in many cases multiple G-quadruplex (GQ) folds. However, it is not clear whether these 2D structures resemble the in vivo folds given the extreme crowding conditions in the trypanosome mitochondrion. This is especially important since RNA editing in vitro has been shown to be sensitive to crowded solvent properties [24].

Here, we analyze the effect(s) of macromolecular crowding on the structure of the mitochondrial RPS12 pre-mRNA as an archetypical example of a mitochondrial transcript in African trypanosomes. We use high molecular mass polyethylene glycol (PEG) as a neutral macromolecular cosolute to mimic intramitochondrial solvent conditions and we monitor the structure of the RPS12 transcript by temperature-dependent UV-spectroscopy and by selective 2′-hydroxyl acylation analyzed by primer extension (SHAPE).

## 2. Materials and Methods

### 2.1. DNA-Cloning and RNA Synthesis

The mitochondrial gene encoding ribosomal protein S12 (RPS12) was PCR-amplified from* T. brucei* Lister 427 genomic DNA (see [25]) using the following DNA-oligonucleotide primers (KpnI and SacI restriction endonuclease recognition/cleavage sites are underlined): RPS12_forw. GGGGTACCCTAATACAC-TTTTGATAACAAACTAAAG; RPS12_rev. CCGAGCTCCCTACCAAACATAAATGAACCTG. The PCR-amplicon was cloned into the KpnI and SacI endonuclease restriction sites of phagemid pBSSKII^−^ (Invitrogen) and transcripts were generated by run off in vitro transcription from linearized plasmid templates using T7-RNA polymerase. RNAs were purified from nonincorporated NTPs by size exclusion chromatography followed by EtOH precipitation. RNA pellets were redissolved in 10 mM Tris/HCl pH 7.5, 1 mM EDTA (TE).

### 2.2. UV-Melting Curves

RPS12 transcripts were dissolved in 0.5x TE pH 7.5 (50 *μ*L), heated to 95°C (2 min), and snap-cooled on ice before the addition of a concentrated folding buffer to yield a final volume of 0.5 mL. Final buffer concentrations were as follows: 5 mM Na-cacodylate, pH 6.8, 70 mM NaCl, and ±2 mM MgCl_2_. Volume-occupied conditions were generated by supplementing the folding buffer with PEG_4000_ at a final concentration of 6% (w/w). This exceeds the polymer crossover concentration (*ϕ*^*∗*^) of PEG_4000_ by a factor of 1.5 and thus represents a highly crowded solvent regime. RNA concentrations were adjusted to *A*_260_ = 0.5. Denaturation/renaturation profiles were measured at 260 nm between 20°C and 95°C at a heating/cooling rate of 1°C/min (data acquisition: 0.3 data points/°C). Melting temperatures (*T*_*m*_) were obtained from the maximum of the first derivatives (*δA*_260_/*δT*) of the melting curves.

### 2.3. SHAPE Modification

The modification reagent 1-methyl-7-nitroisatoic anhydride (1 M7) was synthesized as described [26]. RPS12 pre-mRNA (0.1 *μ*M) was denatured by heating to 95°C (2 min) followed by snap cooling on ice. RNA refolding was achieved by equilibration in 20 mM HEPES, pH 7.5, 30 mM KCl, and ±10 mM MgCl_2_ for 30 min at 27°C, which represents the optimal growth temperature of insect-stage trypanosomes. Crowding conditions were induced by either 6% (w/w) PEG_4000_ (1.5*ϕ*^*∗*^); 12% (w/w) PEG_4000_ (3*ϕ*^*∗*^); or 9% (w/w) PEG_2000_ (1.5*ϕ*^*∗*^). RNA samples were split and treated either with 3.5 mM 1 M7 in DMSO or the same volume of neat DMSO. Modification reactions were quenched after 70 sec by the addition of an equal volume of water. RNAs were recovered by EtOH precipitation and desalted by size exclusion chromatography. The different crowding conditions resulted in nearly identical modification results (see Figure  S1 in Supplementary Material available online at https://doi.org/10.1155/2017/6067345) with Pearson's (*r*) and Spearman's (*ρ*) correlation coefficients ≥ 0.87 and an average standard deviation of ±0.11 SU (SHAPE units).

### 2.4. Reverse Transcription and Data Processing

Equimolar amounts of fluorescently labelled DNA-oligonucleotide primer T3 reverse, 6-FAM/JOE/TAMRA-AATTAACCCTCACTAAA-GGGAAC, were annealed to 1 M7-modified or 1 M7-unmodified RNA samples in 0.25x TE pH 7.5 by heating to 95°C (2 min), cooling to 50°C (10 min), and snap cooling on ice. Reverse transcription was performed in 50 mM Tris/HCl pH 8.3, 75 mM NaCl, 3 mM MgCl_2_, 2.5 mM DTT, 0.25 mM each dNTP, and 0.75 U/*µ*L RiboLock RNase inhibitor (Invitrogen). The reaction was started by prewarming the samples for 1.5 min prior to the addition of 5 U/*µ*L SuperScript III reverse transcriptase (Invitrogen). RPS12 pre-mRNA was reverse-transcribed for 20 min at 40°C. Sequencing reactions were carried out using unmodified RNA, fluorescently labelled DNA-oligonucleotide primer, and ddCTP or ddGTP at a final concentration of 0.125 mM each. Reverse transcription was stopped by snap cooling and the addition of 0.1 volume of 4 M NaOH followed by heating to 95°C (5 min). Samples were pooled, EtOH precipitated, and redissolved in Hi-Di® formamide (ABI/Life technologies) for capillary electrophoresis. Raw electrophoretic traces were analyzed using SHAPEfinder [27] utilizing the box plot approach to determine the number of statistical outliers. Normalized SHAPE reactivities were the result of averaging a minimum of 3 independent experiments.

### 2.5. SHAPE-Directed RNA Folding

Normalized SHAPE reactivities were used as pseudo-Gibbs free energy (Δ*G*) values to guide the folding of the RPS12 pre-mRNA. The minimum free energy (MFE) 2D structure of the RPS12 transcript was generated using the ShapeKnots routine (see [28]) included in RNA structure 5.6 (see [29]) utilizing the default parameters *m* = 1.8 kcal/mol; *b* = 0.6 kcal/mol and *p*1 = 0.35 kcal/mol; *p*2 = 0.65 kcal/mol. Structure comparisons were performed using CircleCompare [29]. MFE structures were compared in terms of their “sensitivity” (sens) and their positive predictive values (ppv): sens = fraction of bp in the reference structure also present in the nonreference structure; ppv = fraction of bp in the nonreference structure also occurring in the reference structure. RPS12 pre-mRNA probed in dilute buffer at physiological that is 10 mM Mg^2+^-ion concentrations served as a reference state if not indicated otherwise.

## 3. Results and Discussion

To study the impact of macromolecular crowding on the structure of mitochondrial pre-mRNAs in* Trypanosoma brucei*, we used the primary transcript of RPS12 as a representative model RNA. The pre-mRNA molecule is 325 nt long. As a pan-edited transcript, it is edited throughout its entire primary sequence with 132 U-nt inserted and 28 U's deleted. The RNA has a G-nt-content of 27% and a purine/pyrimidine (*R*/*Y*) ratio of 1.3. Its experimentally determined minimum free energy (MFE) 2D structure calculates to a Gibbs free energy (Δ*G*) of −152 kcal/mol with a base-paired versus single-stranded nucleotide ratio (*r*_bp/ss_) of 0.62. In addition, the RNA contains a pseudoknot [22]. Since* in cell* structure probing experiments have successfully been performed only with abundant, cytosolic RNAs (see [5, 30–33]), we decided to mimic the crowded, intramitochondrial solvent environment by using a chemically inert, synthetic cosolute such as polyethylene glycol (PEG) [34, 35]. We used two different high molecular mass PEGs with mean molecular masses of 4000 g/mol (PEG_4000_) and 2000 g/mol (PEG_2000_) at concentrations above the polymer crossover concentrations (*ϕ*^*∗*^), which marks the transition from a semidilute to a crowded solvent regime [36–38] (for details, see Materials and Methods).

**Figure 1:**
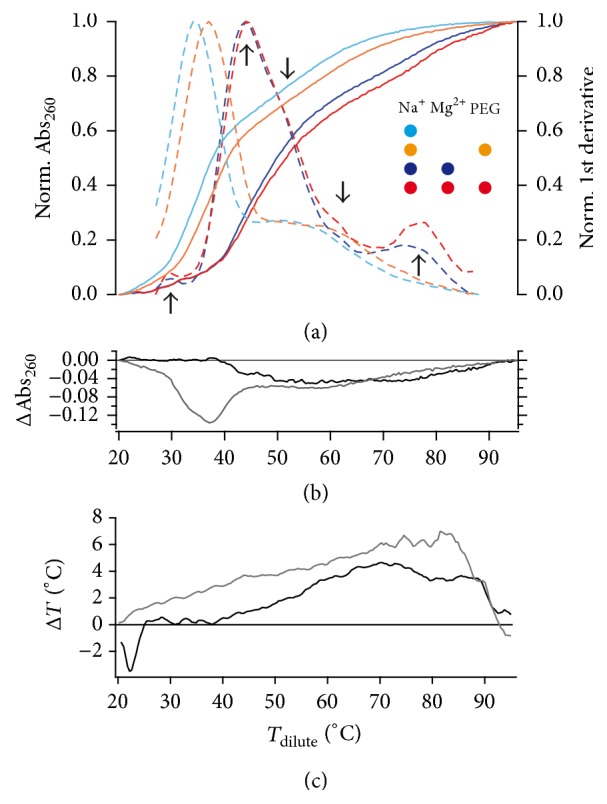
(a) Normalized thermal UV-denaturation profiles of the RPS12 pre-mRNA from* Trypanosoma brucei* (colored, solid lines) at different buffered solvent conditions. Red/orange: crowded solvent conditions (6% (w/w) PEG_4000_). Blue/cyan: dilute solvent conditions. Dark colors: 2 mM Mg^2+^. Bright colors: no Mg^2+^. Dashed traces: normalized 1st derivatives (*δA*_260_/*δT*) of the different melting profiles. Five helix-coil transitions are marked with arrows. (b) Difference (Δ) UV-melting profiles (dilute minus crowded conditions) in the presence/absence of Mg^2+^-cations (black/grey). (c) Shifting of the UV-denaturation profiles to higher temperatures expressed as Δ*T* versus *T* of the dilute condition in presence/absence of Mg^2+^-cations (black/grey).

As a first comparison, we measured UV-melting profiles of the RPS12 pre-mRNA in dilute and crowded solvent conditions. Representative normalized melting profiles and their 1st derivatives (*δA*_260_/*δT*) are shown in Figure 1(a). At dilute buffer conditions (in the presence of 70 mM Na^+^ and 2 mM Mg^2+^), the pre-mRNA displays a complex melting profile with 5 distinct helix/coil transitions: Two dominant transitions with melting midpoints (*T*_*m*_-values) at 46°C and 78°C and three minor transitions at 30°C, 53°C, and 62°C. Almost identical traces were recorded at crowded solvent conditions (Figure 1(a)). As demonstrated in the difference melting-curve in Figure 1(b), the two profiles superimpose perfectly at temperatures ≤40°C and deviate only slightly above 40°C. At that temperature, the “crowded” profile shifts to higher temperature values, however only marginally with Δ*T* of maximally 4°C (Figure 1(c)). This indicates a very small structural stabilization of the transcript at volume-occupied solvent conditions. Since Mg^2+^-cations are known to drive the structural stabilization of RNAs, we wondered whether any larger impact of the crowding agent was masked by the presence of Mg^2+^-cations. As a consequence, we reanalyzed the melting profile of RPS12-RNA in the absence of Mg^2+^. Representative normalized UV-melting curves and their 1st derivatives (*δA*_260_/*δT*) are shown in Figure 1(a). As expected, at dilute solvent conditions, the melting profiles changed drastically: *T*_*m*_ of the main transition shifted by 9.5°C from 44.1°C to 34.6°C and the formerly high temperature transition at 78°C disappeared altogether. However, as before, the PEG-induced stabilization was very small with Δ*T*_*m*_ of 3.4°C for the main transition (Figure 1(a) and Table 1(a)). This demonstrates that the crowding-driven stabilization of RPS12 RNA is by far weaker than the stabilization by divalent cations and that the impact on the overall structure of the transcript is minute.

**Figure 2:**
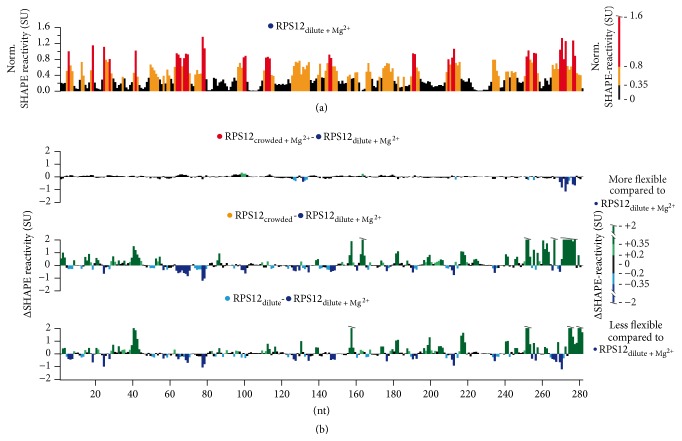
(a) Normalized SHAPE-reactivity profile of the RPS12 pre-mRNA in dilute solvent conditions. Black: low (<0.35 SU); orange: medium (0.35 ≤ SU < 0.8); red: high (≥0.8) normalized SHAPE reactivities. SU: SHAPE-unit. nt: nucleotide(s). (b) Difference (Δ) SHAPE-reactivity profiles of the RPS12 transcript at crowded and/or Mg^2+^-free solvent conditions. Green: nt-positions with increased SHAPE reactivities (dark green: 0.35 ≤ SU ≤ 2; light green: 0.2 ≤ SU < 0.35). Blue: nt-positions with decreased SHAPE reactivities (light blue: −0.2 ≥ SU > −0.35; dark blue: −0.35 ≤ SU ≤ −0.2). Black: nonresponsive nt-positions (−0.2 ≤ SU ≥ 0.2).

As a follow-up of these experiments, we analyzed the effects of macromolecular crowding by probing the structure of the RPS12 RNA with nucleotide resolution. For that we used selective 2′-hydroxyl acylation analyzed by primer extension (SHAPE) [39]. SHAPE monitors the local nucleotide flexibility of conformationally unrestrained nucleotides. In toto 282 nucleotides were interrogated using the four different solvent regimes depicted in Figure 2. The nucleotide flexibility is measured in normalized SHAPE units (SU) and, as expected, the RPS12 transcript displays a complex reactivity pattern including highly reactive (>0.8 SU) and almost unresponsive (<0.35 SU) sequence regions in dilute solvent conditions [22]. Sequence stretches with moderate (0.35 ≤ SU ≤ 0.8) to high flexibility are mostly clustered and alternate with unreactive sequence stretches (Figure 2(a)). About half of the nucleotide positions are inflexible and roughly 10% are highly flexible (>0.8 SU). The same probing experiments were performed at crowded conditions and the SHAPE-reactivity differences in the two solvent regimes were plotted as a difference (Δ_crowded-dilute_) SHAPE profile (Figure 2(b), top panel). The two data sets are characterized by Pearson's (*r*) and Spearman's (*ρ*) correlation coefficients ≥ 0.92 (Tables  S1C/D) indicating that the structure of the RPS12 RNA is nearly identical in the two solvent regimes.

**Figure 3:**
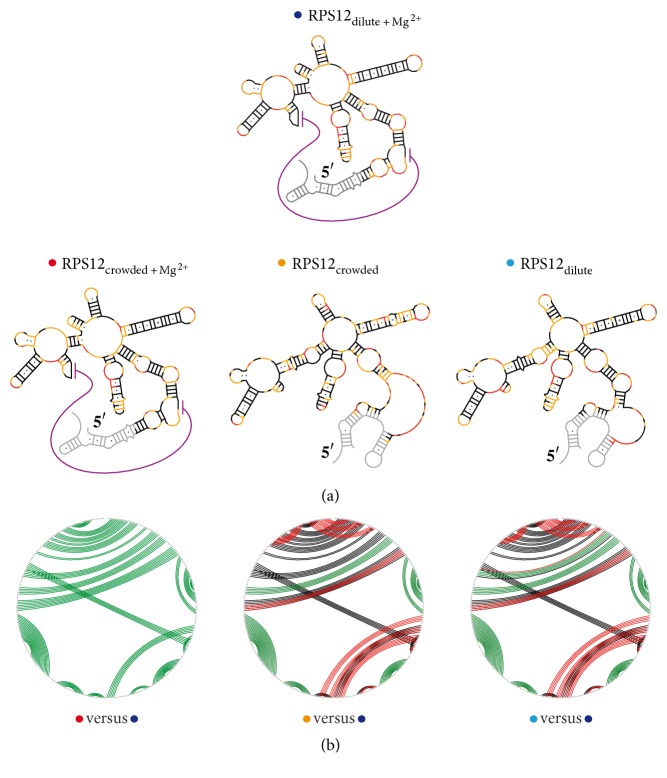
(a) SHAPE-derived minimum free energy (MFE) 2D structures of the RPS12 pre-mRNA: top, at dilute solvent conditions in the presence of 2 mM Mg^2+^; bottom, at crowded solvent conditions in the presence/absence of 2 mM Mg^2+^. (b) Circle-plot comparison of SHAPE-derived 2D structures of the RPS12 pre-mRNA. Base pairs (bp) are shown as colored lines. Green: bp present in both dilute and crowded solvent conditions. Black: bp unique to dilute solvent conditions in the presence of Mg^2+^. Red: bp unique to the specific solvent conditions.

As expected, a comparison of the SHAPE profiles in the absence of Mg^2+^-ions resulted in a different picture. In the absence of the divalent cation, both ΔSHAPE profiles change throughout the entire primary sequence (Figure 2(b), center and bottom panel). About 20% of the nucleotides display reactivity changes >|0.2| SU and an additional 33% show more than a |0.35| SU difference. As a result, the data sets only correlate with correlation coefficients of *r* = 0.27 (*ρ* = 0.35) (crowded-Mg^2+^ versus dilute) and *r* = 0.4 (*ρ* = 0.51) (dilute-Mg^2+^ versus dilute) (Tables  S1C/D and Figure  S2). This behavior translates to the 2D-structure level when the normalized SHAPE reactivities are used as pseudo-free-energy constraints to guide the structure prediction.  Figure 3 shows the pseudoknotted 2D structure of the RPS12 transcript in dilute and crowded solvent conditions. Molecular crowding has no effect on the transcript if Mg^2+^-ions are present. However, since the average and median nucleotide flexibility are slightly decreased at crowded solvent conditions, this results in a decrease of the Gibbs free energy (Δ*G*) of −3.9 kcal/mol (Tables 1(b) and 1(c)). By contrast, in the absence of Mg^2+^-ions, the two structures are characterized by a roughly 15% higher Δ*G* and the absence of the pseudoknot. Sixty percent of the nucleotides retain their structural context at all conditions studied (Figure  S3 and Table  S1).

These results are in line with the published data of Soto et al., 2007, and Tyrrell et al., 2015 (see [3, 40]). As expected, Mg^2+^-ions exert a stabilizing effect on the global fold of RNA molecules, which is reflected in a Δ*T*_*m*_ of 15°C and a ΔΔ*G* of −36 kcal/mol. The contributions of PEG-induced macromolecular crowding to the RNA stability were less pronounced. Crowding conditions caused an increase of the main *T*_*m*_ ≤ 2°C and a decrease in the Gibb's free energy of maximally 4 kcal/mol. Similar trends have been described by Katari et al., 2013, and by Kilburn et al., 2013, [18, 24]. The results obtained by SHAPE- and UV-thermal melting experiments in the absence of Mg^2+^-ions are at a first glance contradictory. The addition of PEG_4000_ resulted in an increase of *T*_*m*_ by about 2°C but had no effect on Δ*G*. Crowding conditions on average even increased the local nucleotide flexibility and had a destabilizing effect on some formerly stable structural elements (Table 1 and Figure 3). This can be explained by the excluded volume effect lowering the degrees of freedom for conformational sampling ultimately trapping the RNA in misfolded states [18, 40]. Alternatively, it is possible that the main transition represents a particularly stable stem structure in the RPS12 pre-mRNA that is further stabilized by PEG. However, this local difference does not manifest itself in the SHAPE-data based, global Δ*G*-calculation. When Mg^2+^ is present, charge compensation by the ion-sphere and/or chelated Mg^2+^-ions lead to an electrostatic collapse of the RNA, thereby generating a highly compact folding state that is further stabilized by the excluded volume effect [3, 4, 17, 41–45]. In the presence and absence of Mg^2+^-ions, the crowding-induced stabilization of the RPS12 pre-mRNA over the entire temperature range most likely results from a perturbed folding landscape due to a reduced conformationally freedom [18]. Based on the data, we conclude that there is no difference in the 2D structure of RPS12 pre-mRNA in dilute and crowded solvent conditions at physiological Mg^2+^-ion concentrations. This is in line with a recent study of Tyrrell et al., 2015 [40]. The authors used SHAPE to compare the folding of the aptamer domain of the adenine riboswitch at dilute, crowded, and in vivo conditions in its free and ligand-bound state. They demonstrated that the 2D structure of the ligand-free aptamer is recapitulated correctly at all conditions despite less pronounced 3D interactions in vitro. Furthermore, ligand addition resulted in the formation of a more compact conformation involving several higher-order 3D interactions that were invariantly mapped in dilute and crowded solvent conditions and inside the cell. Based on our data, we have no evidence for a crowding-induced destabilization of RNA 2D structure in favour of tertiary structure as reported by by Nakano et al., 2009, and Strulson et al., 2014 [46, 47]. However, while the SHAPE data rule out any 2D-structure destabilization, we cannot exclude some effects on the 3D structure of the RNA, which cannot be detected by SHAPE (such as helix packing involving helix/helix interactions).

The RPS12 transcript displays nonvarying local nucleotide flexibilities as well as identical melting profiles at dilute and crowded conditions given that Mg^2+^ is present. This suggests a highly stable and compact structure that obviously is not affected by volume exclusion effects. Thus, we conclude that the in vitro experiments properly reproduce the in vivo situation, at least on the level of RNA secondary structure. Furthermore, we conclude that the described sensitivity of the trypanosomal in vitro RNA editing system [24] cannot be attributed to the structure of the preedited substrate mRNAs, which suggests that the catalytic machinery that is the editosome might be sensitive to volume exclusion conditions. Editosomes are high molecular mass (0.8 MDa), protein-only complexes and the effects of molecular crowding on proteins and protein complexes has been described in detail (reviewed in [48]).

## 4. Conclusions

Mitochondrial transcript maturation in African trypanosomes and other kinetoplastid organisms requires RNA editing in order to generate functional mRNAs. The reaction takes place within the single mitochondrion of trypanosomes, which represents the most crowded environment within eukaryotic cells. RNA substrates of the editing reaction are sequence-deficient preedited mRNAs. Next to the lack of sequence information, they are characterized by the unusual feature of adopting extraordinarily stable secondary structures in dilute solvent conditions. Here we analyzed the effect(s) of macromolecular crowding on the structure of the mitochondrial RPS12 pre-mRNA as an archetypical example of a trypanosome mitochondrial transcript. We demonstrated that macromolecular crowding has no impact on the 2D fold of the RPS12 transcript and we conclude that the minimum free energy (MFE) structure identified in dilute solvent conditions represents a good proxy for the folding of the pre-mRNA in its crowded mitochondrial solvent context. We hypothesize that the data can be extrapolated to all other mitochondrial transcripts in African trypanosomes.

## Supplementary Material

Supplementary Figure S1: SHAPE-reactivity profiles of the RPS12 pre-mRNA in dilute and crowded solvent conditions.
Supplementary Figure S2: Difference (dilute - crowded) SHAPE-reactivity plot of the RPS12 pre-mRNA in the absence of magnesium ions.
Supplementary Figure S3: Summary of the basepairing patterns of the RPS12-transcript at different solvent conditions.
Supplementary Table S1: Statistical comparison of the SHAPE-modification data.

## Figures and Tables

**(a):** 

	Dilute +Mg^2+^	Crowded +Mg^2+^	Dilute	Crowded
*T*_*m*_(°C)_main transition_	44.1	44.6	34.6	37.1

**(b):** 

	Dilute +Mg^2+^	Crowded +Mg^2+^	Dilute	Crowded
Δ*G*_37°C_ (kcal/mol)	−127.4	−131.3	−91.1	−91.8
Fraction ss	0.38	0.38	0.43	0.46
Fraction ds	0.62	0.62	0.57	0.54
Mean reactivity	0.45	0.39	0.57	0.61
Median reactivity	0.35	0.32	0.35	0.37
Pseudoknotted	yes	yes	no	no

**(c):** 

	Crowded + Mg^2+^ versus dilute + Mg^2+^	Dilute versus dilute + Mg^2+^	Crowded versus dilute + Mg^2+^
Δ*T*_*m*_ (°C)	0.5	−9.5	−7.0
ΔΔ*G*_37°C_ (kcal/mol)	−3.9	36.3	35.6
sens (%)	100	56	52
ppv (%)	100	60	58
