# Supplementary material for: The 2D Structure of the T. brucei Preedited RPS12 mRNA Is Not Affected by Macromolecular Crowding
Source: J Nucleic Acids. 2017 Jun 18;2017:6067345. doi: 10.1155/2017/6067345 (PMC5494072; doi:10.1155/2017/6067345)
Supplement: Supplementary file 1 — Supplementary Figure S1: SHAPE-reactivity profiles of the RPS12 pre-mRNA in dilute and crowded solvent conditions. Supplementary Figure S2: Difference (dilute - crowded) SHAPE-reactivity plot of the RPS12 pre-mRNA in the absence of magnesium ions. Supplementary Figure S3: Summary of the basepairing patterns of the RPS12-transcript at different solvent conditions. Supplementary Table S1: Statistical comparison of the SHAPE-modification data. [file 6067345.f1.pdf]

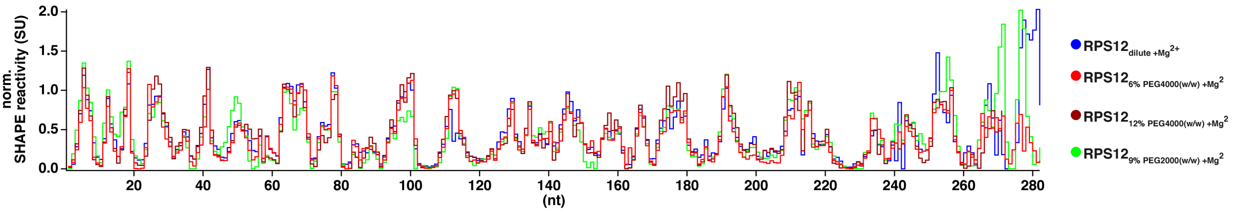

Supplementary Figure S1. Normalized SHAPE-reactivity profiles of the RPS12 pre-mRNA in dilute and three different crowded solvent conditions in the presence of $MgCl_2$ (see legend to the right). The four datasets correlate with a Pearson correlation coefficient of 0.89, a Spearman correlation coefficient of 0.87 and deviate with a mean standard deviation of ±0.11SU. SU=SHAPE-unit.

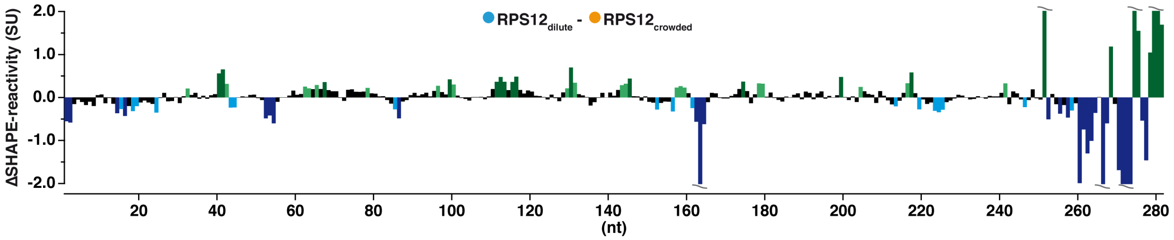

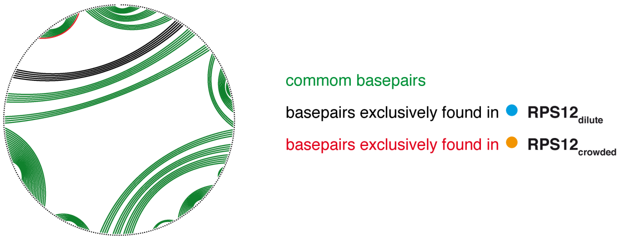

Supplementary Figure S2. Difference (Δ) SHAPE-reactivies of the RPS12 pre-mRNA in dilute and crowded solvent conditions in the absence of Mg^{2+}. SU=SHAPE-unit. The circleplot visualizes common and mutually exclusive basepairs in the two MFE-2D-structures at dilute and crowded conditions.

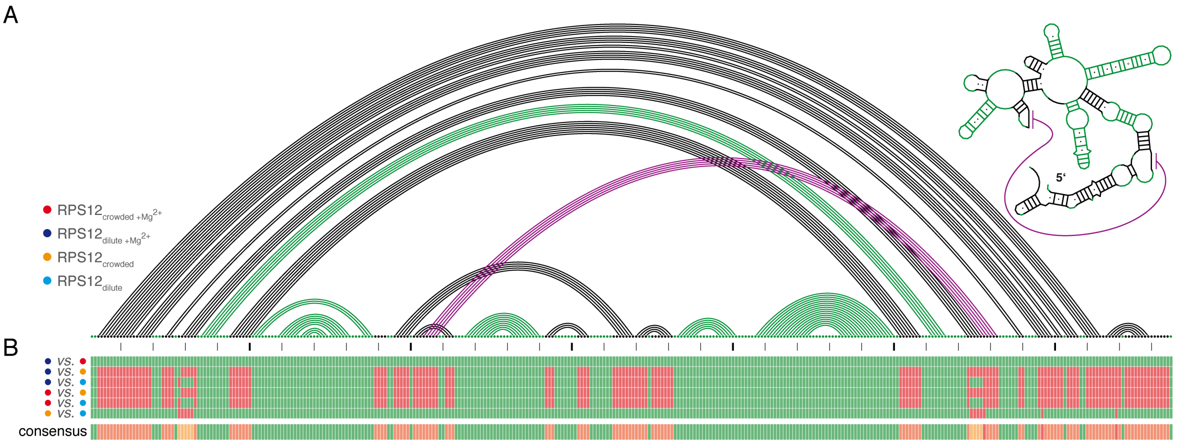

Supplementary Figure S3. A: Arc-representation of the basepairing-pattern in the pre-edited RPS12 mRNA at dilute solvent conditions. Green: invariable basepairs common to all 2D-structures. Black: variable basepairs occurring in a subset of conformations. Purple: RNA pseudoknot (exclusive to the presence of Mg^{2+}). The corresponding 2D-structure is shown in the upper right. B: Pairwise comparison of the different 2D-folds (colour coded as in Fig. 1A): Green: structurally invariant nucleotide position; red: structurally variable nucleotide position (*i.e.* ss to ds; ds to ss or paired to a different basepairing partner). Consensus: Prevalence of every nucleotide position for being either in an invariant or variable structural context. Green: invariant nt; yellow: nt rarely in a variable context; orange: nt predominantely in a variable context; red: nt position predominantely in a variable context compared to dilute buffer conditions in the presence of 10mM Mg^{2+}.

A

sens (%)	dilute $+Mg^{2+}$	crowded $+Mg^{2+}$	dilute	crowded
dilute $+Mg^{2+}$	100	100	56	52
crowded $+Mg^{2+}$		100	56	52
dilute			100	95
crowded				100

B

ppv (%)	dilute $+Mg^{2+}$	crowded $+Mg^{2+}$	dilute	crowded
dilute $+Mg^{2+}$	100	100	60	58
crowded $+Mg^{2+}$		100	60	58
dilute			100	99
crowded				100

C

Pearsons *r*	dilute $+Mg^{2+}$	crowded $+Mg^{2+}$	dilute	crowded
dilute $+Mg^{2+}$	1	0,92	0,40	0,27
crowded $+Mg^{2+}$		1	0,29	0,16
dilute			1	0,74
crowded				1

D

Spearmans ρ	dilute $+Mg^{2+}$	crowded $+Mg^{2+}$	dilute	crowded
dilute $+Mg^{2+}$	1	0,93	0,51	0,35
crowded $+Mg^{2+}$		1	0,46	0,30
dilute			1	0,76
crowded				1

Supplementary Table S1. Solvent condition-dependent comparison of the SHAPE-RNA-modification data of the pre-edited RPS12 transcript on the single nucleotide level (A/B) and on the 2D-structure level (C/D). *r:* Pearson correlation coefficient. *ρ:* Spearman correlation coefficient. Sensitivity (sens) represents the fraction of bp in the reference structure also present in the non-reference structure. Positive predictive value (ppv): fraction of bp in the non-reference structure also occurring in the reference structure.
